# Clinical characteristics and prognosis of myocardial infarction with nonobstructive coronary arteries evaluated by optical coherence tomography

**DOI:** 10.1038/s41598-025-91865-5

**Published:** 2025-03-22

**Authors:** Jie Xia, Chancui Deng, Caifeng Yang, Zaili Lu, Sha Wang, Long Zhang, Zhijiang Liu, Wei Zhang, Ranzun Zhao, Guanxue Xu, Bei Shi

**Affiliations:** 1 Department of Cardiology, Affiliated Hospital of Zunyi Medical University, Zunyi, China; 2 Department of Cardiology, The Fifth Affiliated Hospital of Zunyi Medical University, Zhuhai, China

**Keywords:** Myocardial infarction with Non-Obstructive coronary arteries, Myocardial infarction with coronary artery disease, Optical coherence tomography, Clinical characteristics, Prognosis., Cardiology, Medical research, Pathogenesis

## Abstract

Myocardial infarction with nonobstructive coronary artery (MINOCA) is a heterogeneous disease with different pathophysiological mechanisms and prognosis. In recent years, it has been found that the incidence of major cardiovascular adverse events in MINOCA is similar to that of myocardial infarction with coronary artery disease (MI-CAD), and it is difficult to clarify the pathogenesis of both through coronary angiography (CAG). Therefore, the aim of this study is to investigate the clinical features, plaque characteristics and prognosis of patients with MINOCA and MI-CAD through optical coherence tomography (OCT). A total of 553 culprit lesions from AMI patients who underwent CAG and OCT were retrospectively analysed. Patients were subsequently divided into two groups: the MINOCA group and the MI-CAD group. The clinical characteristics, plaque characteristics and prognosis of the two groups were compared. The primary endpoint was defined as a composite of major adverse cardiac events (MACE), including cardiac death, non-fatal myocardial infarction, target lesion revascularization, stroke, and rehospitalisation for angina or heart failure. (1) Patients with MINOCA exhibited a lower incidence of ST-segment elevated myocardial infarction (STEMI) and a less frequent history of combined drug-eluting stent (DES) compared to those with MI-CAD. Additionally, they demonstrated lower levels of low density lipoprotein cholesterol (LDL-C), total cholesterol (TC), triglycerides (TG), peak troponin T (peak TnT) and peak creatine kinase (peak CK). The MINOCA group had more lesions in the left anterior descending (LAD) and fewer in the left circumflex (LCX). Additionally, they demonstrated a lower prevalence of multibranch vasculopathy and a diminished post-discharge use of aspirin, P2Y12 receptor inhibitors, beta-blockers, angiotensin converting enzyme inhibitor/angiotensin receptor blockers (ACEI/ARBs), and a higher proportion of conservative treatment compared to DES. The frequency of plaque rupture, calcified plaque, cholesterol crystals, macrophages infiltration, microvessels, thin-cap fibroatheroma (TCFA), and thrombus were found to be lower in the MINOCA group than in the MI-CAD group, with these differences being statistically significant (*P* < 0.05); (2) No significant difference was observed in the incidence of MACE at 30-days and 1 year between patients in the MINOCA and MI-CAD groups (*P* > 0.05). Compared with MI-CAD patients, MINOCA patients had fewer high-risk plaques on OCT and were more likely to be treated conservatively, with lower rates of stenting and less post-discharge pharmacological treatment. Both groups had similar rates of MACE at 30-day and 1 year, highlighting the importance of individualising treatment for MINOCA patients. Patients with MINOCA who develop MACE are more likely to exhibit high-risk OCT plaque features, with macrophage infiltration identified as an independent risk factor. OCT plaque features such as plaque rupture, plaque erosion, cholesterol crystals, macrophages, microvessels, TCFA may have played different roles in the progression of the two groups of patients.

**Supplementary Information:**

The online version contains supplementary material available at 10.1038/s41598-025-91865-5.

## Introduction

Myocardial infarction with nonobstructive coronary artery (MINOCA) is a complex and varied disease, approximately 6% of patients with acute myocardial infarction (AMI) are diagnosed with MINOCA^1,2^. Given that the aetiology of MINOCA differs from that of myocardial infarction with obstructive coronary artery disease (MI-CAD), the treatment strategy and prognosis diverge considerably. Optical coherence tomography (OCT), an advanced biomedical imaging technique, can provide insight into the underlying causes of coronary artery disease, assess the integrity of atherosclerotic plaque, and identify features of atherosclerosis^3,4^. The objective of this study was to conduct a systematic analysis of patients with MINOCA using the OCT technique. The aim was to reveal their clinical features, plaque characteristics, treatment strategies and prognosis within 30-days and 1 year. This will contribute to a more comprehensive understanding of the pathological mechanisms of MINOCA and provide an important basis for the early identification of patients with acute myocardial infarction and the development of individualised therapeutic regimens, which will lead to improved prognosis and reduced mortality.

## Objects and methods

### Objects

**Fig. 1:**
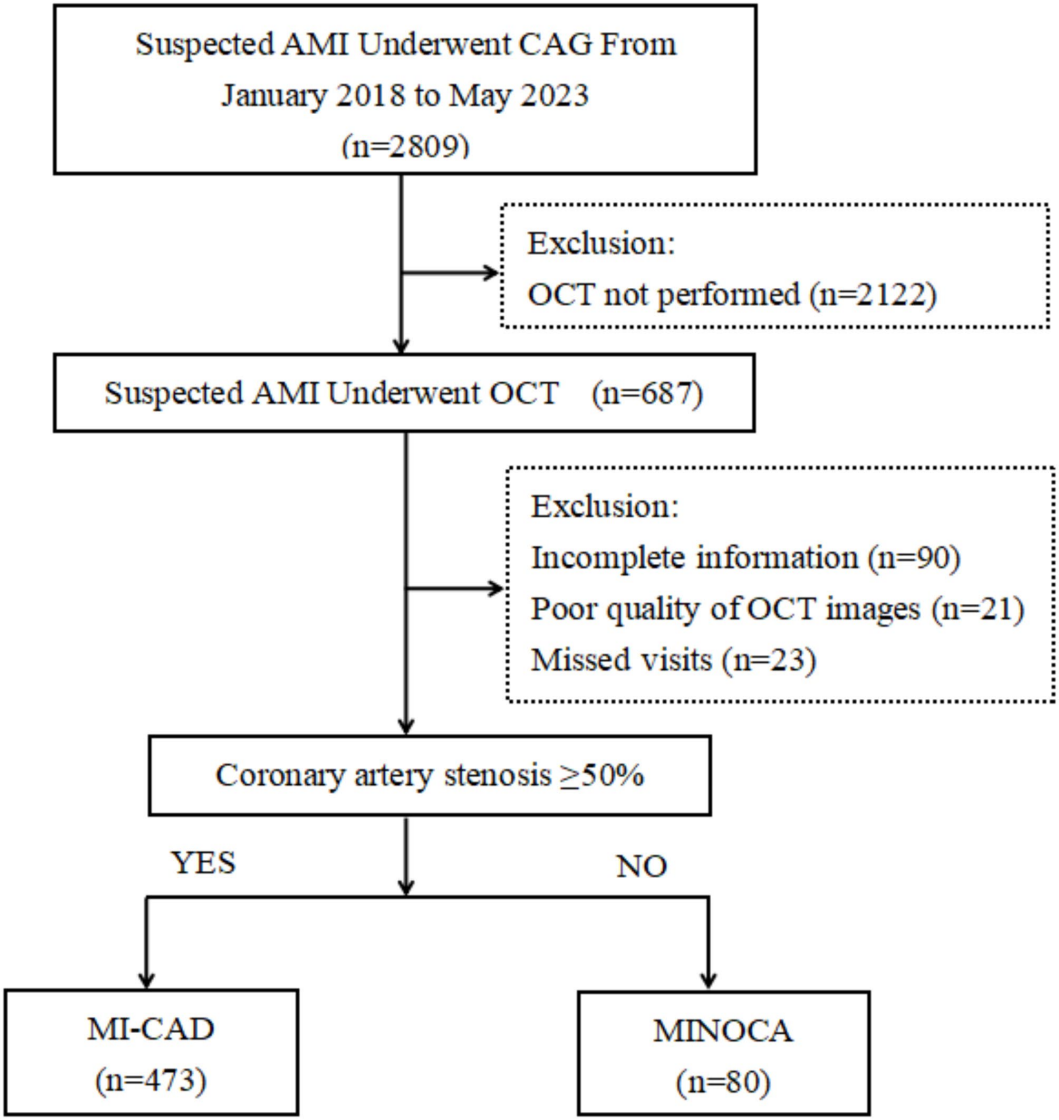
Study flowchart. AMI = Acute myocardial infarction; CAG = Coronary angiography; MI-CAD = Myocardial infarction with obstructive coronary artery disease; MINOCA = Myocardial infarction with nonobstructive coronary artery; OCT = Optical coherence tomography.

A retrospective analysis of 687 patients with AMI from January 2018 to May 2023 at the Affiliated Hospital of Zunyi Medical College was conducted. All patients underwent coronary angiography (CAG) and OCT of culprit lesions at our centre. The definition of an “culprit lesion” was as follows: an interrupted or hedonic flow with visible thrombus on coronary angiography, along with distal supply of side branches; severe stenosis with slow flow and no TIMI flow grade 3; TIMI flow grade 3 but visible thrombus shadows at the lesion site; and severe stenosis with no visible thrombus shadows. The lesions were identified by experienced cardiologists through a combination of electrocardiography, imaging, physiological assessment, and an assessment of the severity of the stenosis. Ultimately, 553 patients with AMI who fulfilled the requisite inclusion and exclusion criteria were included in the analysis (Fig. 1). Based on the results of coronary angiography, the patients were finally divided into two groups: the MINOCA group (*n* = 80) and the MI-CAD group (*n* = 473). The study was approved by the Ethics Committee of the Affiliated Hospital of Zunyi Medical College and conducted in accordance with the Declaration of Helsinki, confirm that all studies were conducted in accordance with relevant guidelines and regulations, and that all patients participating in this study provided informed consent.

#### Inclusion criteria

(1) All subjects accepted CAG and OCT examination and completed the process of signing the informed consent; (2) Complete clinical data; (3) Age ≥ 18 years old; (4) The diagnostic criteria of MINOCA refer to the statement on the diagnosis and management of MINOCA issued by the American Heart Association in 2019^5^: ① The patient must meet the diagnostic criteria for AMI. ② Coronary angiography must show non-obstructive coronary arteries without ≥ 50% coronary stenosis of any major epicardial vessel. ③ There must be no other alternative diagnosis that causes this clinical manifestation, such as pulmonary embolism or myocarditis. The diagnostic criteria for MI-CAD refer to the 4th edition of myocardial infarction^6^. The diagnostic criteria for MI-CAD refer to clinical evidence of elevated or decreased levels of myocardial injury markers (mainly TNT) exceeding the upper limit of the reference value by at least 99%, accompanied by acute myocardial ischemia (as long as one condition is met), including: (a) Symptoms of myocardial ischaemia; (b) New ischaemic electrocardiographic changes; (c) The emergence of new Q-waves; (d) Imaging evidence of new loss of viable myocardium or abnormalities of ventricular wall phase motion; (e) Coronary arteriography or intracavitary imaging or autopsy confirming coronary thrombosis^6^.

#### Exclusion criteria

(1) Incomplete case data; (2) poor OCT image quality; (3) missed visits.

### Data collection

#### Baseline data and angiographic analysis

Two trained technicians collected demographic and clinical characteristics of patients by reviewing their hospital records, which were blinded to the purpose of the study. Smokers were defined as those with a smoking history of more than six months and who had not quit smoking at the time of the survey. Diabetes mellitus was defined as a self-reported history of diabetes mellitus or use of antidiabetic medication, or a fasting blood glucose level ≥ 7.0 mmol/L and a non-fasting blood glucose level > 11.1 mmol/L. Blood samples were taken in the fasting state and specific parameters included blood glucose, creatinine, lipids, brain natriuretic peptide (BNP), cardiac enzymes and blood counts, and CAG was performed using quantitative coronary artery analysis (QCA) software (Artis VC21C, Siemens AG, Berlin and Munich, Germany) for offline analysis.

#### Optical coherence tomography image acquisition

**Fig. 2:**
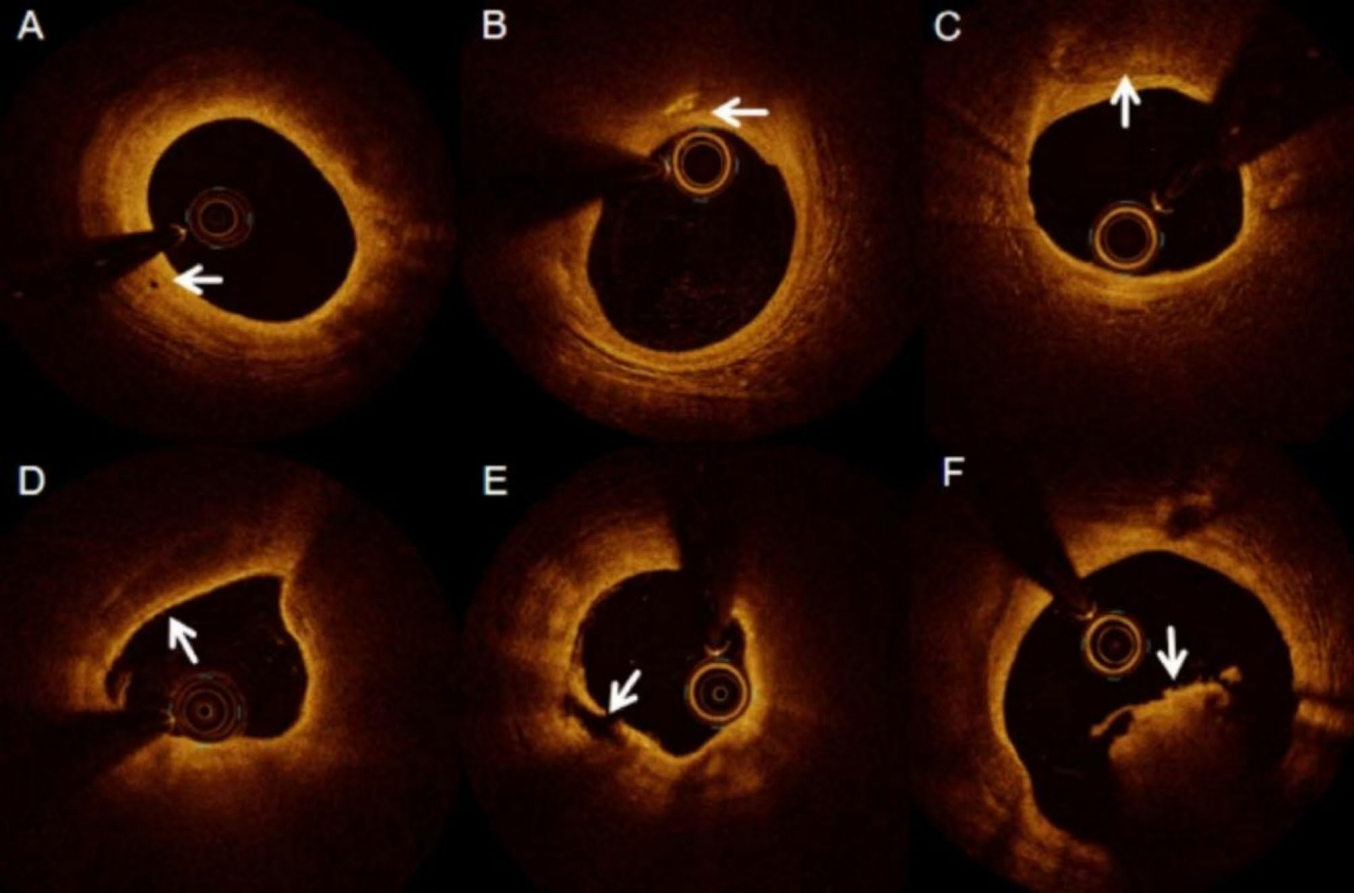
Optical coherence tomography (OCT) images of different types of plaques. A: Neovascularization; B: Crystal; C: calcified plaque; D: Thin cap fibroatheroma (TCFA); E: Plaque rupture; F: Thrombus.

Optical coherence tomography images were obtained using a frequency domain OCT system, specifically the C7XR/ILUMIEN/ILUMIEN OPTIS intracavitary imaging system (St. Jude Medical, St. Paul, MN, USA). The imaging procedure was based on the recommendations of a previous expert consensus^7^. Briefly, a guidewire is advanced to the distal coronary artery to locate the target lesion. After precise localisation, a contrast agent was injected into the coronary artery using a catheter. At the same time, the OCT system is activated to acquire images. During OCT image acquisition, the contrast agent is used to clear the vessel. All OCT images are digitally stored for later offline analysis. All OCT images were graded by two independent observers. If the two observers disagreed, a third observer was consulted to reach a consensus. Representative OCT images are shown in Fig. 2.

####  Definitions of relevant indicators

**Table:** 

ACEF score	The formula is: ACEF = age/left ventricular ejection fraction + 1 (if blood creatinine > 176.8 µmol-L-1 or > 2.0 mg-dL-1)^8^
NLR	Neutrophil-to-lymphocyte ratio^9^
Definition of OCT-related indicators	
Plaque rupture	Rupture of the fibrous cap covering the lipid plaque, i.e. the fibrous cap is discontinuous and a cavity is formed within the plaque^10^
Plaque erosion	The presence of thrombus, irregular luminal surfaces and an intact fibrous cap were observed^10^
TCFA	The fibre cap thickness is less than or equal to 65 μm at its thinnest point^11^
Calcified plaque	Well-defined, inhomogeneous low-signal areas^11^
Cholesterol crystals	Linear areas of high signal within plaques
Macrophages	Highly reflective, strongly attenuating point or strip-like structures, and often forming radial light shadows after point-like areas of high signal^11^
Microvessels	For the explicit identification of signal difference nulls across multiple consecutive frames^11^
Thrombus	Irregular mass protruding from the lumen and discordant with the wall. Red thrombus was defined as high backscatter and high attenuation, and white thrombus was defined as homogeneous low backscatter and low attenuation^11^
Major adverse cardiac events	Cardiac death, non-fatal myocardial infarction, target lesion revascularization, stroke, re-hospitalisation for angina or heart failure
Cardiac death	Death due to malignant arrhythmia, acute myocardial infarction, heart failure or other heart disease^12^
Non-fatal myocardial infarction	In addition to the typical symptoms of myocardial ischaemia, cardiac biomarkers or electrocardiographic dynamic changes are positive^12^
Target lesion revascularization	Restoration of blood perfusion to narrowed or occluded cardiovascular vessels by means of drugs or surgery to restore blood supply to the corresponding ischaemic organs
Stroke	Ischaemic cerebral infarction due to thrombus or embolic obstruction^12^
Re-hospitalisation for angina or heart failure.	Re-admission was due to clinical signs such as worsening of chest pain symptoms compared to the previous one or the development of heart failure. The diagnosis of heart failure was made according to the recent ESC guidelines for the diagnosis and treatment of acute and chronic heart failure^13^

### Statistical treatment

The statistical analysis software employed was SPSS 29.0 (IBM SPSS Statistics Professional Software). The one-sample Kolmogorov-Smirnov test was employed to ascertain whether the data exhibited a normal distribution. In the case of normally distributed measures, these were expressed as mean ± standard deviation, and comparisons between two groups were made using the independent samples t-test. In contrast, non-normally distributed measures were expressed as median [M(Q1, Q3)], and comparisons between groups were made using the Mann-Whitney U test. In the case of count data, the rates were expressed, and comparisons between groups were made using the χ² test or Fisher’s exact probability method. The two groups were subjected to survival analysis using the Kaplan-Meier method, with statistical significance considered at a level of *P* < 0.05.

##  Results

### Comparison of baseline data between MINOCA and MI-CAD groups

The present study included a total of 553 patients, comprising 473 patients with MI-CAD and 80 patients with MINOCA. A comparison of the two groups revealed that patients in the MINOCA group had experienced fewer ST-segment elevated myocardial infarction (STEMI) patients, had a shorter history of combined percutaneous coronary intervention (PCI), and exhibited lower levels of low-density lipoprotein cholesterol (LDL-C), total cholesterol (TC), triglycerides (TG), and peak troponin T (peak TnT) and peak creatine kinase (peak CK). Additionally, they had more lesions in the left anterior descending (LAD) and fewer in the left circumflex (LCX). Furthermore, there were fewer multibranch vasculopathies, less use of aspirin, P2Y12 receptor inhibitors, β-blockers, and angiotensin converting enzyme inhibitor/angiotensin receptor blockers (ACEI/ARBs) after discharge, and more patients treated conservatively and fewer who underwent DES, with statistically significant differences (*P* < 0.05). As illustrated in Table 1.

**Table 1:** Comparison of baseline data between the two groups of patients.

	ALL (*n* = 553)	MI-CAD (*n* = 473)	MINOCA (*n* = 80)	*P* value
General information				
Age (years)	57.00 (50.00, 68.00)	57.00 (50.00, 68.00)	56.50 (48.25, 66.75)	0.556
Male, n (%)	448 (81.1)	387 (81.8)	61(76.3)	0.240
Smoking, n (%)	324 (58.6)	282 (59.6)	42 (52.5)	0.232
Hypertension, n (%)	269 (48.6)	231 (48.8)	38 (47.5)	0.825
Diabetes, n (%)	93 (16.8)	81 (17.1)	12 (15.0)	0.638
CKD, n (%)	16 (2.9)	15 (3.2)	1 (1.3)	0.489
Hyperlipidemia, n (%)	93 (16.8)	81(17.1)	12 (15.0)	0.638
History of previous heart failure, n (%)	16 (2.9)	16 (3.4)	1 (1.3)	0.489
History of previous PCI, n (%)	108 (19.6)	108 (22.8)	2 (2.5)	<0.001
STEMI, n (%)	350 (63.3)	325 (68.7)	25 (31.3)	<0.001
HDL-C (mmol/L)	1.08 (0.94, 1.24)	1.08 (0.94, 1.24)	1.11 (0.96, 1.26)	0.790
LDL-C (mmol/L)	3.01 ± 0.86	3.04 ± 0.87	2.81 ± 0.75	0.027
Cr (µmol/L)	78.00 (67.50, 91.00)	78.00 (68.00, 91.00)	78.50 (67.00, 91.00)	0.834
LVEF (%)	54.00 (44.00, 60.00)	54.00 (44.00, 59.50)	55.00 (44.00, 61.00)	0.273
ACEF	1.14 (0.93, 1.40)	1.15 (0.95, 1.39)	1.10 (0.86, 1.42)	0.290
Blood glucose (mmol/l)	6.18 (5.09, 9.04)	6.23(5.07,9.04)	5.97(5.29,8.47)	0.876
TC (mmol/L)	4.98 (4.06, 5.71)	5.02 (4.12, 5.82)	4.60 (3.85, 5.41)	0.013
TG (mmol/L)	1.68 (1.11, 2.68)	1.72 (1.13, 2.75)	1.495 (0.98, 2.23)	0.040
BNP (pg/ml)	453.70 (128.25, 1372.00)	463.00 (130.50, 1362.00)	400.00 (102.03, 1501.67)	0.628
Peak TNT	684.00 (130.75, 2167.00)	790.60 (176.20, 2209.00)	213.80 (19.80, 1834.00)	<0.001
Peak CK	356.00 (138.00, 1027.00)	391.00 (153.50, 1125.00)	241.50 (96.00,788.00)	0.010
WBC (10^9^/l)	8.91 (6.90, 11.01)	9.11 (7.11, 11.09)	7.83 (6.31, 10.49)	0.115
N (10^9^/l)	6.44 (4.47, 9.08)	6.63 (4.49, 9.10)	5.61 (4.26, 8.92)	0.440
L (10^9^/l)	1.46 (1.07, 1.83)	1.49 (1.08, 1.87)	1.30 (1.00, 1.71)	0.062
NLR	4.42 (2.79, 7.54)	4.36 (2.83, 7.54)	4.49 (2.67, 9.54)	0.796
Culprit vessel				
LM, n (%)	4(0.7)	4(0.8)	0(0)	1.000
LAD, n (%)	324 (58.6)	266 (56.2)	58 (72.5)	0.006
RCA, n (%)	133 (24.1)	117 (24.7)	16 (20.0)	0.359
LCX, n (%)	71 (12.8)	67 (14.2)	4 (5.0)	0.023
Multiple, n (%)	24 (5.1)	24 (5.1)	0 (0)	0.036
Medications at discharge				
Aspirin, n (%)	511 (92.4)	446 (94.3)	65 (81.3)	<0.001
P2Y12 inhibitor, n (%)	478 (86.4)	435 (92.0)	43 (53.8)	<0.001
Statin, n (%)	531 (96.4)	457 (96.6)	76 (95.0)	0.513
β-Blocker, n (%)	403 (72.9)	359 (75.9)	44 (55.0)	<0.001
ACEI/ARB, n (%)	390 (70.5)	343 (72.5)	47 (58.8)	0.013
Processing method				
DES, n (%)	412(74.5)	409(86.5)	3(3.8)	<0.001
DCB, n (%)	54 (9.8)	51 (10.8)	3 (3.8)	0.056
PTCA, n (%)	47 (8.5)	39 (8.2)	8 (10.0)	0.603
Thrombus aspiration, n (%)	41 (7.4)	32 (6.8)	9 (11.3)	0.157
Conservative treatment with medication, n (%)	80 (14.5)	19 (4.0)	61 (76.3)	<0.001

Data are expressed as the median [interquartile range], mean ± SD, or n (%). ACEF=Age, creatinine, and ejection fraction score; ACEI/ARB = Angiotensin converting enzyme inhibito/Angiotensin receptor blockers; BNP = Brain natriuretic peptide; CKD = Chronic kidney disease; Cr = Creatinine; DES = Drug-eluting stent; DCB = Drug-Coated; HDL-C = High-density lipoprotein cholesterol; LDL-C = Low-density lipoprotein cholesterol; LVEF = Lventricular Ejection Fraction; LM = Left main; LAD = Left anterior descending; LCX = Left circumflex; L = Lymphocyte; MI-CAD = Myocardial infarction with obstructive coronary artery disease; MINOCA = Myocardial infarction with nonobstructive coronary artery; N = Neutrophil; NLR = Neutrophil–lymphocyte ratio; PTCA = Percutaneous transluminal coronary angioplasty; RCA = Right coronary artery; STEMI = ST-segment elevation myocardial infarction‌; TC = Total cholesterol; TG = Triglyceride; TNT = Troponin; WBC = White blood cell.

### Comparison of plaque characteristics under OCT between MINOCA and MI-CAD groups

**Fig. 3:**
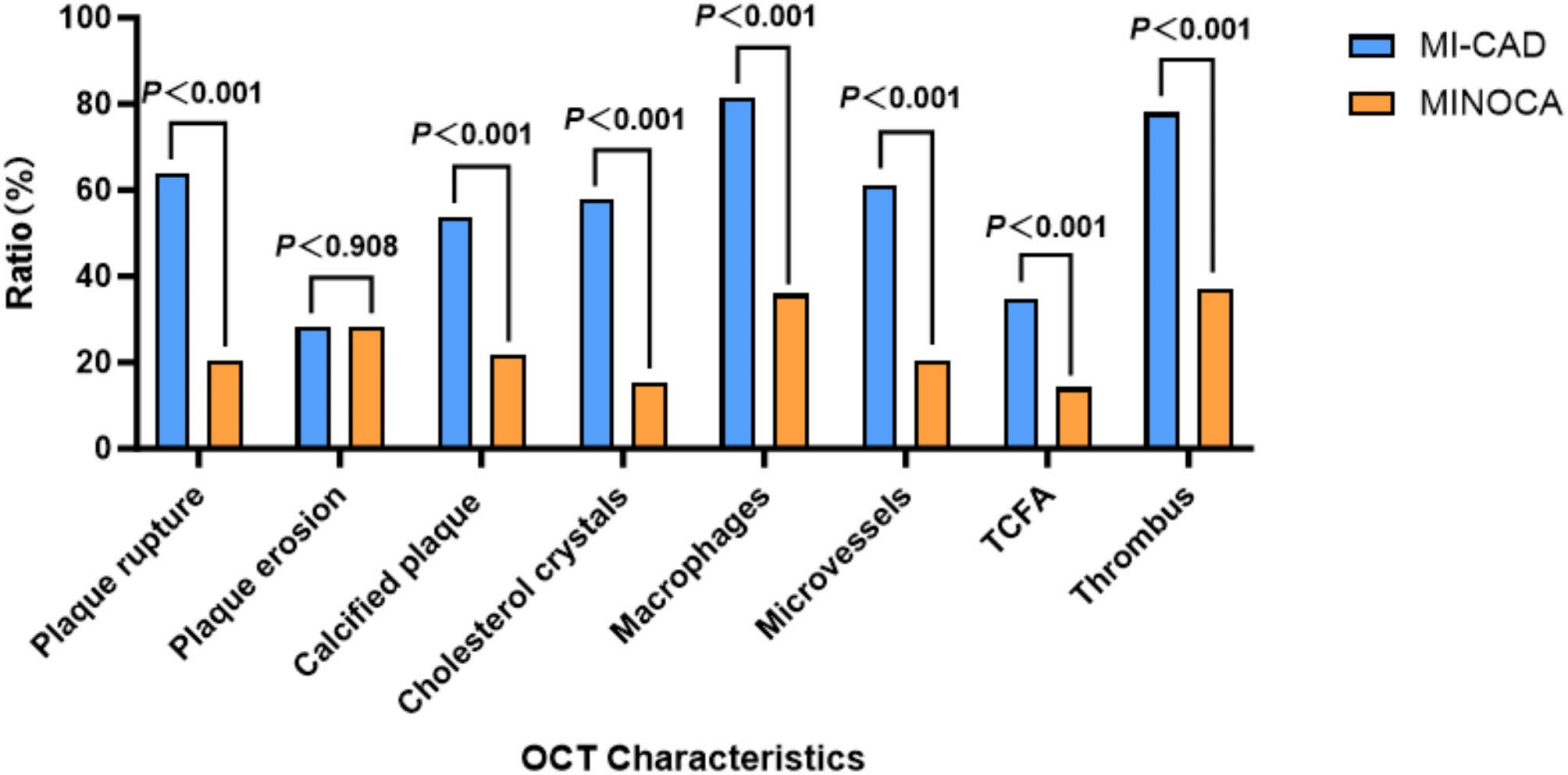
Comparison of plaque features under OCT between the two groups of patients. MI-CAD = Myocardial infarction with obstructive coronary artery disease; MINOCA = Myocardial infarction with nonobstructive coronary artery; OCT= Optical coherence tomography; TCFA = Thin-cap fibroatheroma.

The frequency of plaque rupture, calcified plaque, cholesterol crystals, macrophages, microvessels, thin-cap fibroatheroma (TCFA) and thrombus in patients in the MINOCA group were found to be lower than those in the MI-CAD group. These differences were all statistically significant (*P* < 0.05). (Supplementary Tables 1 and Fig. 3.)

### Comparison of major adverse cardiovascular events between MINOCA group and MI-CAD group

The incidence of major adverse cardiovascular events within 30-day and 1 year was comparable between patients in the MINOCA and MI-CAD groups (*P* > 0.05), as demonstrated in Tables 2 and 3.

**Table 2:** Comparison of major adverse cardiovascular events in 30-day between the two groups of patients.

	ALL (*n* = 553)	MI-CAD (*n* = 473)	MINOCA (*n* = 80)	*P* value
Major cardiovascular events	71 (12.8)	63 (13.3)	8 (10)	0.412
Cardiac death, n (%)	12 (2.2)	12 (2.5)	0 (0)	0.231
Non-fatal MI, n (%)	1 (0.2)	1 (0.2)	0 (0)	1.000
TLR, n (%)	8 (1.4)	8 (1.7)	0 (0)	0.61
Stroke, n (%)	3 (0.5)	3 (0.6)	0 (0)	1.000
Re-hospitalisation for heart failure or angina, n (%)	54 (9.8)	46 (9.7)	8 (10)	0.939

MI-CAD = Myocardial infarction with obstructive coronary artery disease; MINOCA = Myocardial infarction with nonobstructive coronary artery; MI=Myocardial infarction; TLR = Target lesion revascularization.

**Table 3:** Comparison of major adverse cardiovascular events in 1 year between the two groups of patients.

	ALL (*n* = 553)	MI-CAD (*n* = 473)	MINOCA (*n* = 80)	*P* value
Major cardiovascular events	133 (24.1)	114 (24.1)	19 (23.8)	0.946
Cardiac death, n (%)	13 (2.4)	12 (2.5)	1 (1.3)	0.704
Non-fatal MI, n (%)	7 (1.3)	6 (1.3)	1 (1.3)	1.000
TLR, n (%)	21 (3.8)	20 (4.2)	1 (1.3)	0.197
Stroke, n (%)	9 (1.6)	8 (1.7)	1 (1.3)	1.000
Re-hospitalisation for heart failure or angina, n (%)	94 (17)	79 (16.7)	15 (18.8)	0.652

MI-CAD = Myocardial infarction with obstructive coronary artery disease; MINOCA = Myocardial infarction with nonobstructive coronary artery; MI=Myocardial infarction; TLR = Target lesion revascularization.

### Comparison of 1-year survival analysis between MINOCA group and MI-CAD group

**Fig. 4:**
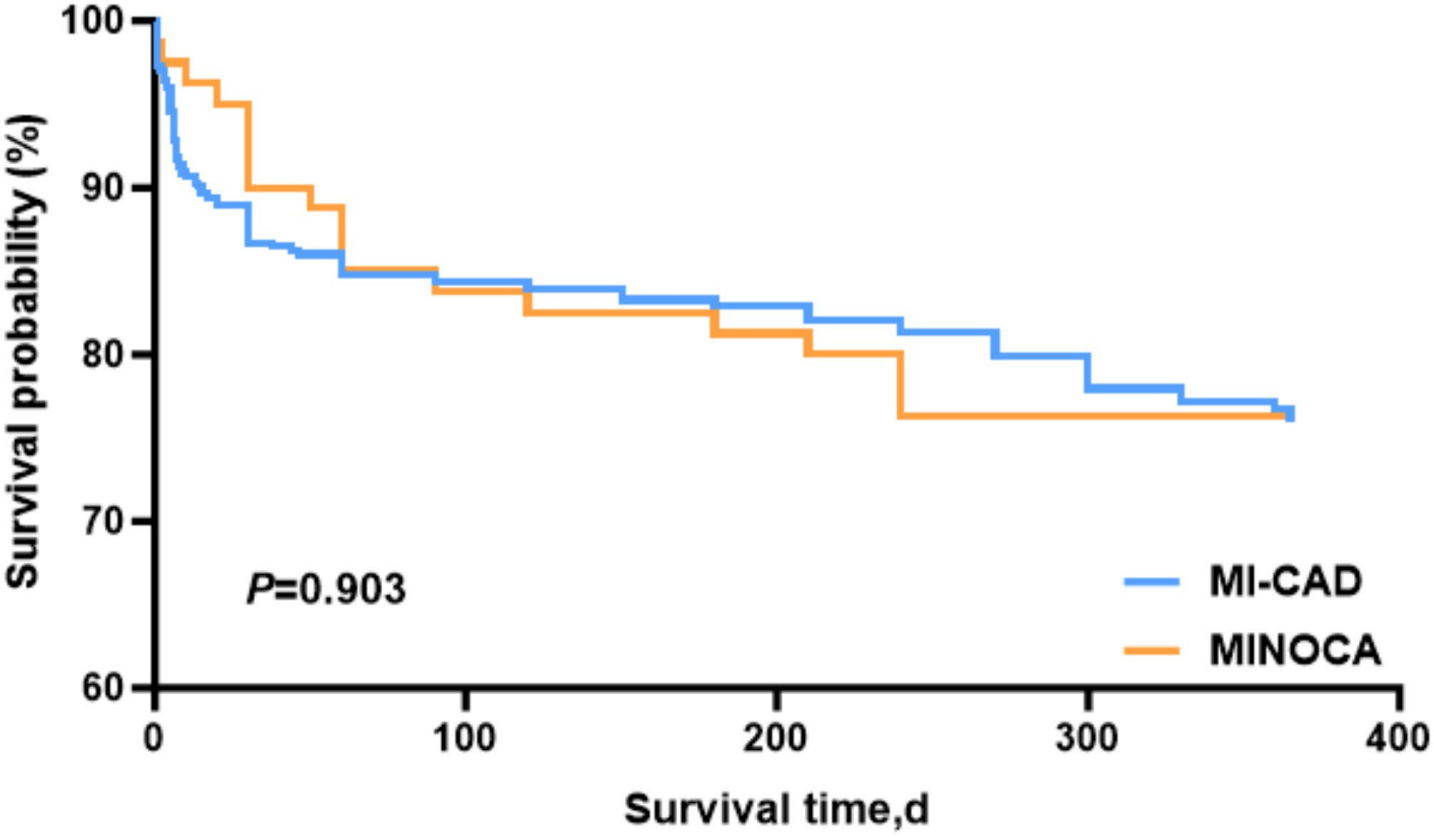
Comparison of survival analysis at 1 year in MINOCA and MI-CAD groups. MI-CAD = Myocardial infarction with obstructive coronary artery disease; MINOCA = Myocardial infarction with nonobstructive coronary artery;

As illustrated in Fig. 4, the Kaplan-Meier curve demonstrates that the early MI-CAD group exhibited a faster decline than the MINOCA group, suggesting that major adverse cardiac events (MACE) was more likely to occur in the short term. Conversely, the late MI-CAD group demonstrated a gradual decline and exhibited overlap with the MINOCA group, indicating that there was no significant difference in the incidence of MACE between the two during the 1-year follow-up period (*P* > 0.05).

## Discussion

### Clinical characteristics of patients with MINOCA

Previous studies have shown that MINOCA patients account for 6–15% of acute myocardial infarction cases^1^. Patients with MINOCA accounted for 14.5% (80/553) of all AMI patients in the study, indicating that the prevalence of patients with MINOCA is not low. Among the 80 patients with MINOCA included in this study, 61 were male and 19 were female, with a male-to-female ratio of approximately 3:1, suggesting that the incidence rate is higher in males, which is largely consistent with the findings of previous studies in China. In contrast, the results of relevant foreign studies^14,15^, such as Safdar et al.^2^, demonstrated that the risk of MINOCA in women is five times higher than that in men. This may be attributable to geographical and ethnic disparities, in addition to potential research bias. In this study, the incidence of STEMI was lower in patients with MINOCA compared to those with MI-CAD, which is consistent with previous findings^16,17^. It is proposed that the degree of coronary obstruction is low in patients with MINOCA, and ST-segment elevation is frequently attributable to complete occlusion of the coronary arteries and the formation of a limited number of collateral circulations. Consequently, STEMI is more prevalent in MI-CAD. In this study, a history of combined DES was uncommon among patients with MINOCA, indicating that the proportion of exposure to various risk factors was lower among patients with MINOCA compared to patients with MI-CAD. Prior research has also indicated a reduced incidence of hyperlipidaemia in patients with MINOCA in comparison to those with MI-CAD, with a comparable pattern observed in other coronary risk factors^2^. In the present study, LDL-C, cholesterol, and triglyceride levels were observed to be lower in the MINOCA patients than in the MI-CAD group. This finding is consistent with the results of previous studies and suggests that the onset of MINOCA may not be exclusively due to thrombosis and atherosclerosis induced by traditional risk factors. Jedrychowska et al.^18^ demonstrated that troponin is a predictor of MACE in patients with MINOCA. Troponin, as a quantitative marker of the extent of cardiomyocyte damage, indirectly reflects the severity of coronary obstruction. In this study, peak TNT and peak CK levels were observed to be lower in the MINOCA patients than in the MI-CAD group, which suggests that myocardial damage was less severe in the MINOCA patients than in the MI-CAD group. Troponin is released in response to a number of factors, including increased left ventricular wall pressure, inflammatory factor injury, massive catecholamine release, oxidative stress, and direct mechanical injury to cardiomyocytes^19^. Accordingly, an elevated troponin value is indicative of an increased probability of MI-CAD. The data presented in this study demonstrated that lesions in patients with MINOCA were more frequently observed in the LAD, less frequently observed in the LCX, and less frequently observed in cases where multibranch vasculopathy was present (*P* < 0.05), which is consistent with the findings of previous studies. This indicates that in patients with MINOCA, as in other AMIs, infarcts are frequently observed in the region innervated by the anterior descending branch of the left coronary artery, followed by the right coronary artery, and subsequently in the area innervated by the gyratory branch of the left coronary artery. It has been demonstrated that the number of involved coronary artery branches is an independent risk factor for determining the prognosis of patients with coronary artery disease^20^. The involvement of a greater number of vascular branches is associated with a more severe patient condition and a poorer prognosis^21,22^. Consequently, patients with MINOCA are less likely to present with multibranch vasculopathy. In this study, we also observed that a greater proportion of patients with MINOCA were treated conservatively, and a smaller number underwent DES (*P* < 0.05). This may be attributed to the fact that patients with MINOCA often present with minimal luminal stenosis and relatively mild lesions compared to those with MI-CAD, which may be managed without intervention. Conversely, this may be attributed to the fact that some medical practitioners lack a comprehensive understanding of the pathophysiological mechanisms underlying MINOCA and the characteristics of plaques. Consequently, they may be unable to provide tailored treatment regimens. Previous studies have demonstrated that the proportion of patients with MINOCA who were discharged with statins, ACEI/ARBs, β-blockers and dual antiplatelet therapy was 84.5%, 64.1%, 83.4% and 66.4%, respectively. Furthermore, these studies have indicated that statins and ACEIs/ARBs may confer long-term benefits to patients with MINOCA, whereas the benefit of β-blockers remains uncertain. However, the use of dual antiplatelet therapy did not yield any significant benefits in these patients^23^. The data from this study demonstrated that patients with MINOCA were discharged with a reduced dosage of aspirin, P2Y12 receptor inhibitors, β-blockers, and ACEIs/ARBs (*P* < 0.05), while the proportion of statin lipid-lowering therapy did not differ significantly from that observed in MI-CAD patients. The results demonstrate that in recent years, cardiovascular physicians have progressively acquired greater knowledge of individualised drug therapy for patients with MINOCA. Furthermore, the treatment of MINOCA is not solely based on thrombotic events. In order to enhance the long-term prognosis of this group of patients, it is essential to reinforce the use of ACEI/ARB in patients with MINOCA.

### Plaque characteristics in patients with MINOCA

Pathological evidence suggests that plaques with TCFA, larger lipid cores and rich in macrophages, microvessels and severe calcified plaque are more prone to rupture and are often referred to as vulnerable plaques. OCT is currently the most valuable intravascular imaging technique for identifying vulnerable plaques, especially for diagnosing plaque instability. Pinilla-Echeverri N et al.^24^ in their subgroup study found that obstructive lesions had more vulnerable plaque features (lipid-rich, TCFA, macrophages, cholesterol crystals) compared to non-obstructive lesions. One study^25^ highlighted that speckle calcified plaque is frequently associated with elevated lipid indices, thin fibrous caps, microvessels formation, and other characteristics. Consequently, speckle calcified plaque can be employed as a marker of vulnerable plaques under OCT. In a separate study, Napodano and colleagues^26^ observed a correlation between an elevated thrombus load and a larger infarct size. In this study, patients in the MINOCA group exhibited lower levels of plaque rupture, calcified plaque, cholesterol crystals, macrophages, microvessels, TCFA, and thrombus compared to those in the MI-CAD group. These findings are consistent with the results of previous studies in this field. It is evident that the atherosclerotic plaque at the infarct site was more stable in MINOCA patients compared with MI-CAD patients. Furthermore, it was more favourable to MI-CAD patients when combined with multiple high-risk OCT plaque features.

### Prognosis of MINOCA patients

The prognosis of MINOCA is a topic of ongoing debate in the medical community. A systematic review^2^ reported an in-hospital mortality rate of 0.9% and a 12-month mortality rate of 4.7%, which is lower compared to MI-CAD. In contrast, a study by Magnani G et al.^27^ demonstrated that patients with MINOCA exhibited a relatively elevated risk of major cardiovascular events, approaching that observed in patients with MI-CAD. The SWEDEHEART registry report^23^ indicated that the incidence of MACE in patients with MINOCA can reach as high as 30.7% over a mean follow-up period of 12 months. In this study, a total of 553 patients with AMI were observed for a period of one year, with a median follow-up time of 365 days. Of the total number of patients, 133 patients (24.1%) had 144 major adverse cardiovascular events during the 12 months of follow-up, including 13 cardiac deaths (2.4%), 7 nonfatal myocardial infarctions (1.3%), 21 TLRs (3.8%), 9 strokes (1.6%), and 94 rehospitalisations due to angina pectoris or heart failure (17%). Patients with MI-CAD experienced 114 MACE (24.1%), 12 cardiac deaths (2.5%), 6 nonfatal myocardial infarctions (1.3%), 20 TLRs (4.2%), 8 strokes (1.7%), and 79 rehospitalisations for angina or heart failure (16.7%). MINOCA patients had 19 MACE (23.8%), 1 cardiac death (1.3%), 1 nonfatal myocardial infarction (1.3%), 1 TLR (1.3%), 1 stroke (1.3%), and 15 rehospitalisations for heart failure or angina (18.8%). It can be seen that there is no significant difference in clinical outcomes at 12 months between MI-CAD and MINOCA patients (*P* > 0.05). In addition, this study also analyzed the 30-day clinical outcomes of the two groups of patients and concluded that the 30-day and 1-year mortality rates of MINOCA patients were similar to those of MI-CAD patients and their quality of life was comparable, which is consistent with the VIRGO study^28^. It suggests that although patients with MINOCA do not have significant coronary stenosis, it still poses a great danger to patients, which is consistent with the results of several studies at home and abroad^29–31^. However, this study found that although there was no significant difference in the number of patients rehospitalised for angina or heart failure between the two groups at 1-year follow-up, it was higher in the MINOCA group (18.8%) than in the MI-CAD group (16.7%), which on the one hand, may be due to the lack of clarity in the diagnostic etiology of MINOCA and the lack of uniformity in the treatment method. Since non-atherosclerotic causes of MINOCA often have no stenosis in the lumen, they go unnoticed by patients, resulting in poor compliance and high rates of rehospitalisation. On the other hand, probably because this study is a single-centre, retrospective study with a small sample size and a short follow-up period, it is still necessary to further expand the sample size and extend the time frame of the study to more scientifically assess the value of OCT in the follow-up of patients with acute heart attack. In addition, the low percentage of females in our cohort may be due to the large proportion of males among AMI patients treated at our centre or the low percentage of females receiving CAG and OCT. Given that single-centre retrospective studies may have potential selection bias, future large-sample, prospective studies are needed to further validate these results. In addition, this study showed that plaque rupture, plaque erosion, cholesterol crystals, macrophages, microvessels, and TCFA were risk factors for the development of MACE in patients with MINOCA by univariate regression analysis (Supplementary Table 2). This is in line with the findings of the series by Prati^32^, Dai^33^, and Taruya’s^34^ team. Plaque instability is the most important cause of MACE in patients with AMI, and OCT is currently the most valuable intravascular imaging technique for identifying plaque instability. The above study used OCT to analyze plaque properties and found that when plaques had high-risk plaque characteristics (TCFA, lipid arc > 180°, MLA < 3.5 mm^2^, and macrophage infiltration), they had a 7.54-fold elevated risk of a primary endpoint event, and that culprit vessels with cholesterol crystals exhibited more fragile plaque characteristics (greater lipid loading, macrophage infiltration, plaque rupture), and and there was a strong correlation between increased trophoblasts and plaque vulnerability. Therefore, it is important to be vigilant for MACE when patients with MINOCA present with high-risk OCT plaque features. Furthermore, macrophages were identified as an independent risk factor for the development of MACE in patients with MINOCA, following the exclusion of univariate-related confounders. This increased the likelihood of MACE by 7.344-fold within 1 year in patients with MINOCA (Supplementary Table 3). This is consistent with the CLIMA study^35^, which found that the greater the macrophage angle (> 67°) and the shallower the location (< 0.12 mm), the higher the incidence of the composite end point of cardiac death, myocardial infarction, or target vessel revascularization. When macrophages are found in atherosclerotic plaques in patients with MINOCA, it is important to be vigilant for MACE. Finally, in this study, a subgroup analysis of OCT plaque features was also performed on patients in the MINOCA and MI-CAD groups, and it was found that OCT features such as plaque rupture, plaque erosion, cholesterol crystals, macrophages, microvessels, and TCFA showed significant interactions between the outcomes of patients with MI-CAD and MINOCA (Supplementary Fig. 1.), suggesting that these features may play a different roles. However, the sample size of this study was relatively small, so performing subgroup analyses may have resulted in statistically unstable or misleading results. Further studies are needed in the future to fully understand this interaction.

### Advantages

In this study, we compared the clinical features, plaque characteristics, and prognosis of patients with MINOCA and MI-CAD using OCT, analyzed the risk factors of MINOCA patients who developed MACE by COX, and also explored the correlation between OCT features and MACE in both groups. The findings suggest a potential association between high-risk plaques under OCT and the development of MACE in patients with MINOCA. Therefore, the use of OCT for evaluation in patients with MINOCA allows for the early detection of high-risk plaques in patients with MINOCA, which is valuable for individualized treatment and prognosis, thus contributing to clinical decision-making and improving patients’ quality of life.

### Limitations

This study was a retrospective single-center survey with a relatively small sample size, and the retrospective nature of the study may have introduced uncontrolled confounding factors that could have affected the validity of the results. Therefore, the findings must be validated in further large-scale, prospective, multicenter studies; The use of OCT intracavitary imaging is limited by the clinical characteristics of the patient and the location of the lesion, and only includes AMI patients who have undergone OCT and have complete clinical and OCT data, which may lead to selection bias. In addition, although subgroup analyses were performed in this study, because the study sample size was relatively small and the basis for subgroup delineation was not yet adequate, performing subgroup analyses may have introduced statistically unstable or misleading results.

## Conclusions

Compared with MI-CAD patients, MINOCA patients had fewer high-risk plaques on OCT and were more likely to be treated conservatively, with lower rates of stenting and less post-discharge pharmacological treatment. Both groups had similar rates of MACE at 30-day and 1 year, highlighting the importance of individualising treatment for MINOCA patients. Patients with MINOCA who develop MACE are more likely to exhibit high-risk OCT plaque features, with macrophage infiltration identified as an independent risk factor. OCT plaque features such as plaque rupture, plaque erosion, cholesterol crystals, macrophages, microvessels, TCFA may have played different roles in the progression of the two groups of patients.

## Electronic supplementary material

Below is the link to the electronic supplementary material.

Supplementary Material 1

## Data Availability

The data that support the findings of this study are available from the corresponding author upon reasonable request.

## Abbreviations

ACEF: Age, creatinine, and ejection fraction score
ACEI/ARBs: Angiotensin converting enzyme inhibitor/angiotensin receptor blockers
AMI: Acute myocardial infarction
BNP: B*rain natriuretic peptide*
CAG: Coronary angiography
CKD: Chronic kidney disease
CK: Creatine kinase
Cr: Creatinine
DES: Drug-eluting stent
DCB: Drug-coated balloon
HDL-C: High-density lipoprotein cholesterol
LDL-C: Low-density lipoprotein cholesterol
LVEF: Lventricular ejection fraction
LM: Left main
LAD: Left anterior descending
LCX: Left circumflex
L: L*ymphocyte*
MACE: Major adverse cardiac events
MI-CAD: Myocardial infarction with obstructive coronary artery disease
MINOCA: Myocardial infarction with nonobstructive coronary artery
N: Neutrophil
NLR: N*eutrophil–lymphocyte ratio*
OCT: Optical coherence tomography
PTCA: *Percutaneous transluminal coronary angioplasty*
RCA: Right coronary artery
STEMI: ST-segment elevation myocardial infarction
TLR: Target lesion revascularization
TC: T*otal cholesterol*
TG: Triglyceride
TCFA: Thin-cap fibroatheroma
TNT: Troponin T
WBC: White blood cell
