# Supplementary material for: Clinical characteristics and prognosis of myocardial infarction with nonobstructive coronary arteries evaluated by optical coherence tomography
Source: Sci Rep. 2025 Mar 22;15:9962. doi: 10.1038/s41598-025-91865-5 (PMC11929927; doi:10.1038/s41598-025-91865-5)
Supplement: Supplementary file 1 — Supplementary Material 1 [file 41598_2025_91865_MOESM1_ESM.docx]

**SUPPLEMENTARY MATERIALS**

**Clinical Characteristics and Prognosis of Myocardial Infarction with Nonobstructive Coronary Arteries Evaluated by Optical Coherence Tomography**

**Supplementary Table 1. Comparison of plaque characteristics under OCT between the two groups of patients**

|  | | **ALL (n=553)** | **MI-CAD (n=473)** | **MINOCA (n=80)** | ***P* value** |
| --- | --- | --- | --- | --- | --- |
|  |  |  |  |  |  |
| OCT characteristics | |  |  |  |  |
|  | Plaque rupture, n (%) | 319 (57.7) | 302 (63.8) | 17 (21.3) | ＜0.001 |
|  | Plaque erosion, n (%) | 156 (28.1) | 133 (28.1) | 23 (28.7) | 0.908 |
|  | Calcified plaque, n (%) | 273 (49.4) | 254 (53.7) | 19 (23.8) | ＜0.001 |
|  | Cholesterol crystalsls, n (%) | 286 (51.7) | 273 (57.7) | 13 (16.4) | ＜0.001 |
|  | Macrophages, n (%) | 413 (75.0) | 385 (81.4) | 28 (35.0) | ＜0.001 |
|  | Microvessels, n (%) | 306 (55.3) | 289 (61.1) | 17 (21.3) | ＜0.001 |
|  | TCFA, n (%) | 167 (32.0) | 165 (34.9) | 12 (15.0) | ＜0.001 |
|  | Thrombus, n (%) | 399 (72.2) | 369 (78.0) | 30 (37.5) | ＜0.001 |

MI-CAD=Myocardial infarction with obstructive coronary artery disease; MINOCA=Myocardial infarction with nonobstructive coronary artery; OCT＝ Optical coherence tomography；TCFA＝Thin-cap fibroatheroma.

**Supplementary Table 2. Univariate Cox regression analysis of the occurrence of MACE in patients with MINOCA**

|  | | **HR** | **95%CI** | ***P* value** |
| --- | --- | --- | --- | --- |
| OCT characteristics | |  |  |  |
|  | Plaque rupture | 3.438 | 1.376-8.588 | 0.008 |
|  | Plaque erosion | 2.588 | 1.049-6.382 | 0.039 |
|  | Calcified plaque | 1.704 | 0.647-4.489 | 0.281 |
|  | Cholesterol crystals | 5.088 | 2.017-12.837 | ＜0.001 |
|  | Macrophages | 6.675 | 2.395-18.606 | ＜0.001 |
|  | Microvessels | 4.298 | 1.739-10.622 | 0.002 |
|  | TCFA | 3.550 | 1.347-9.357 | 0.010 |
|  | Thrombus | 2.130 | 0.865-5.245 | 0.100 |

OCT＝ Optical coherence tomography；TCFA＝Thin-cap fibroatheroma.

**Supplementary Table 3. Multivariate COX regression analysis of the occurrence of MACE in patients with MINOCA**

|  | | **HR** | **95%CI** | ***P* value** |
| --- | --- | --- | --- | --- |
| OCT characteristics | |  |  |  |
|  | Plaque rupture | 0.872 | 0.238-3.191 | 0.836 |
|  | Plaque erosion | 0.882 | 0.278-2.798 | 0.831 |
|  | Cholesterol crystals | 2.437 | 0.738-8.050 | 0.144 |
|  | Macrophages | 7.344 | 1.479-36.469 | 0.015 |
|  | Microvessels | 1.019 | 0.257-4.032 | 0.979 |
|  | TCFA | 2.148 | 0.685-6.733 | 0.190 |
|  | Thrombus | 0.512 | 0.148-1.776 | 0.292 |

OCT＝ Optical coherence tomography；TCFA＝Thin-cap fibroatheroma.


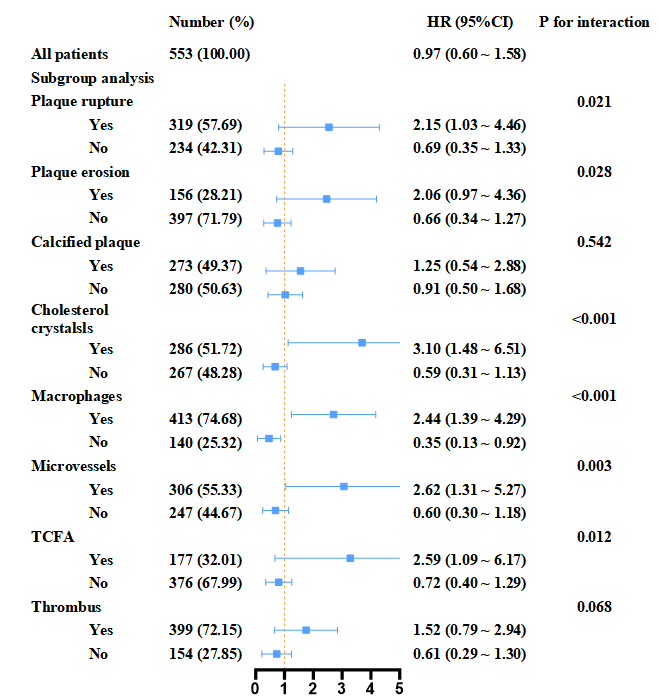


**Supplementary Figure 1. Subgroup analysis and forest map**

TCFA＝Thin-cap fibroatheroma.
